# More Purpose in Life and Less Novelty Seeking Predict Improvements in Self-Compassion During a Mindfulness-Based Intervention: The EXMIND Study

**DOI:** 10.3389/fpsyt.2020.00252

**Published:** 2020-04-03

**Authors:** Mari Akase, Takeshi Terao, Nobuko Kawano, Akari Sakai, Koji Hatano, Masanao Shirahama, Hirofumi Hirakawa, Kentaro Kohno, Nobuyoshi Ishii

**Affiliations:** ^1^ Department of Neuropsychiatry, Faculty of Medicine, Oita University, Yufu, Japan; ^2^ Department of Psychology, Faculty of Welfare and Health Sciences, Oita University, Oita, Japan

**Keywords:** psychotherapy, mindfulness, self-compassion, novelty seeking, purpose in life

## Abstract

**Objectives:**

Recently, a 4-week mindfulness-based intervention followed by a 4-week existential approach was found to be as effective for increasing self-compassion as an 8-week mindfulness-based intervention. The purpose of the present study was to identify the factors that predicted change in self-compassion during the 8-week mindfulness-based intervention.

**Methods:**

Fifty-seven of the 61 completers of the 8-week mindfulness-based intervention provided baseline, 4-week, and 8-week self-compassion scale scores. The mean age of the 47 females and 10 males was 49.6 years. Pearson’s correlation coefficients were generated on the associations between the change of total self-compassion scale scores from baseline to 8 weeks with age; gender; and the baseline scores on the Temperament Evaluation of Memphis, Pisa and San Diego Auto-questionnaire, Temperament and Character Inventory (TCI), Mini-Mental State Examination, Japanese Adult Reading Test, Young Mania Rating Scale, Hamilton Rating Scale for Depression, Parental Bonding Instrument, and purpose in life (PIL). Multiple regression analysis was performed to identify the predictors of the change in total self-compassion scale scores.

**Results:**

Novelty seeking (TCI) was significantly and negatively associated with the change in total self-compassion scale scores, whereas the PIL scores were significantly and positively associated with the change in total self-compassion scale scores. Novelty seeking was not significantly associated with baseline, 4-week, or 8-week total self-compassion scale scores, whereas the PIL scores were significantly and positively associated with baseline, 4-week, and 8-week total self-compassion scale scores. The limitation of the present study was a relatively small number of subjects which deterred a more sophisticated analysis of the pathways involved.

**Conclusions:**

The present findings suggest that more PIL and less novelty seeking predict improvements in self-compassion during mindfulness-based interventions, although novelty seeking might substantially predict the improvement but self-compassion scale and PIL might somewhat conceptually overlap.

## Introduction

Recently, we conducted a randomized controlled trial to examine whether a mindfulness-based intervention (MBI) and an existential approach could be sequentially combined and whether they operated antagonistically or cooperatively. In that study, apparently healthy participants were first assigned to a wait-listed group and, then, they were randomized into the 8-week MBI group or the 4-week MBI followed by 4-week existential approach (EXMIND) group. The main outcome variable was self-compassion measured on a self-compassion scale with total scores assessed at 0, 4, and 8 weeks during the interventions or waiting period. Both intervention groups had significantly increased total self-compassion scale scores compared to those of the waiting group, suggesting that EXMIND was not antagonistic and might have cooperative effects with the mindfulness approach, and that EXMIND might be a useful treatment (1).

As we described in the previous paper (1), mindfulness is the process of acknowledging subjective experience, and it has emotional regulation process, which is broader than attentional control. Although attention is a key component of mindfulness practice, mindfulness also incorporates an openness to experience, which reflects a non-judgmental acceptance strongly linked to improved health. MBIs can be traced back to the late 1970s. A mindfulness-based stress reduction (MBSR) program was begun in 1979 in the basement of the University of Massachusetts Medical Center (2), where Kabat-Zinn (3) initially reported that mindfulness meditation produced significant pain reduction in chronic pain patients. Since then, numerous treatment protocols based on MBSR, such as mindfulness-based cognitive therapy (MBCT), have been developed. MBSR and MBCT are two of the most widely used MBIs. Their positive effects on mental health and quality of life have been reported in diverse clinical and non-clinical populations (4–6).

In our study (1), during the first half (4 weeks), both the MBI and EXMIND groups attended the same MBI sessions which included raisin exercise, mindful breathing, body scan, walking meditation, and sitting meditation. Participants received explanatory notes at every session. Homework including both formal and informal training was encouraged for participants to train themselves for mindfulness reskilling. During the second half (4 weeks), the MBI group continued to perform meditation and homework, and thereafter discussed their experiences during individual instruction; at session 5, sitting meditation and individual instructions for an openness to experience and non-judgmental acceptance during meditation; at session 6, sitting meditation and individual instructions for a gap between thought and fact, making a meditation plan, and recording achievement as homework; at session 7, sitting meditation and individual instructions for widening opportunities for selection, and resolution of the gap between meditation plan and achievement; and at session 8, sitting meditation, individual instructions for utilizing mindfulness for daily life, and seeking triggers to remind of mindfulness. Therefore, we believe that the MBI in tour study resemble MBSR and MBCT in many ways.

Nonetheless, the factors that predict MBI have not been determined. For example, comorbid personality disorders might predict non-completion of MBCT (7) whereas openness and agreeableness might predict greater use of MBSR (8). Men or highly extraverted individuals experience considerably lower MBCT effectiveness (9), and the same author found that neuroticism predicted benefits of MBSR (10). High educational attainment predicted a decrease in depressive symptoms by MBCT (11). Greater depressive severity was associated with higher levels of mind-wandering (in contrast to mindfulness) and lower levels of self-compassion (12). The interaction between low conscientiousness and MBSR and that between high neuroticism and MBSR each predicted significantly lower levels of depression and distress at 12-month follow-up compared to women who were higher in conscientiousness or lower in neuroticism (13). The practice of mindfulness meditation was positively related to openness and extraversion and negatively related to neuroticism and conscientiousness (14). Both attachment anxiety and attachment avoidance were negatively associated with total mindfulness (15). Although MBCT was associated with signiﬁcant increases in trait positive affect and momentary positive cognition (16), in a systematic review, two found no association between mindfulness interventions and cognitive function, two found improvement that was not sustained at the follow-up, and another two found sustained improvement at 2- or 6-months (17). We could not find any report showing cognition as a predictor of MBI responses. As for purpose in life (PIL; or life’s meaning), patients’ search for life’s meaning was the only significant predictor of willingness to participate in MBSR in cancer patients (18). Also, cancer patients endorsing higher levels of dispositional mindfulness were more likely to pay attention to positive experiences, a tendency which was associated with positive reappraisal of stressful life events. Moreover, patients who engaged in more frequent positive reappraisal had a greater sense of meaning in life (19). Finally, a meta-analysis showed that small and statistically significant pooled effects of MBIs on combined measures of psychological distress were found at post-intervention and follow-up, and that larger effects of MBIs on psychological distress were found in studies (a) adhering to the original MBI manuals, (b) with younger patients, (c) with passive control conditions, and (d) shorter time to follow-up (20). Overall, the literature suggests that people who are open to new experiences and extraverted, have a positive mood and have a greater PIL engage well in mindfulness behavioral intervention.

Therefore, it seems possible that gender, temperament, personality, education, cognition, mental state, parental bonding, and PIL may affect the effects of MBI. Considering these possibilities, in the present study, it was hypothesized that the effects of MBI may be associated with these factors.

The present study was performed to test the above hypothesis. In other words, in the previous study (1), MBI improved self-compassion of apparently healthy volunteers in the form of a group therapy at the first half and an individual therapy at the last half in a city hall. Therefore, the rationale of the present study was the effects of MBI on self-compassion in apparently healthy individuals and the purpose of the study was to determine which type of apparently healthy people can increase self-compassion *via* MBI.

## Materials and Methods

This study used data derived from our previous study (1) that, in brief, was a randomized controlled trial at the Oita city hall (Horuto-Hall Oita), Japan. Participants were recruited in Oct 1, 2016 and June 30, 2018. The Institutional Review Board of Oita University Faculty of Medicine approved the trial on Sep 14, 2016 (number B16-023). All participants provided informed written consent. The protocols of the primary study conformed to the provisions of the Declaration of Helsinki and were approved by the Oita University Ethics Committee. Because the purpose of the present study was one of the purposes of the primary study, permission to use the secondary data use was assumed.

In advance, to detect changes in self-compassion scale scores, J score by MBI or EXMIND, considering the effect size of MBI may be 0.5 at a *p*-value of less than 0.05 with 80% power, 60 participants per group were estimated to be needed, allowing for 20% loss to follow-up (1). However, the effect size of 0.5 might have been overestimated. Fifty-seven of the 61 completers in the MBI group had baseline, 4-week, and 8-week self-compassion scale scores (21). The mean age of the 47 females and 10 males was 49.6 years. We investigated change over time in the total self-compassion scale scores using repeated analysis of variance (ANOVA) tests and, when statistically significant, Tukey’s *post hoc* comparison tests were performed. Statistical relationships were tested (Pearson’s correlations) between the difference in total self-compassion scale scores from baseline to 8 weeks (Δself-compassion scale scores) and age; gender; the baseline scores of the Temperament Evaluation of Memphis, Pisa and San Diego Auto-questionnaire (TEMPS-A) (22), Temperament and Character Inventory (TCI) (23), Mini-Mental State Examination (MMSE) (24), Japanese Adult Reading Test (JART) (25), Young Mania Rating Scale (YMRS) (26), Hamilton Rating Scale for Depression (HRSD) (27), Parental Bonding Instrument (PBI) (28), and PIL (29), all of which were performed just before the MBI intervention. In addition, because high educational attainment predicted a decrease in depressive symptoms by MBCT (11), we investigated the association of educational level (1 = *high school or less*, 2 = *college*, and 3 = *university or more*) with Δself-compassion scale scores. Finally, a multiple regression analysis was performed to identify the predictors of the improvement in total self-compassion scale scores.

## Results

**Figure 1:**
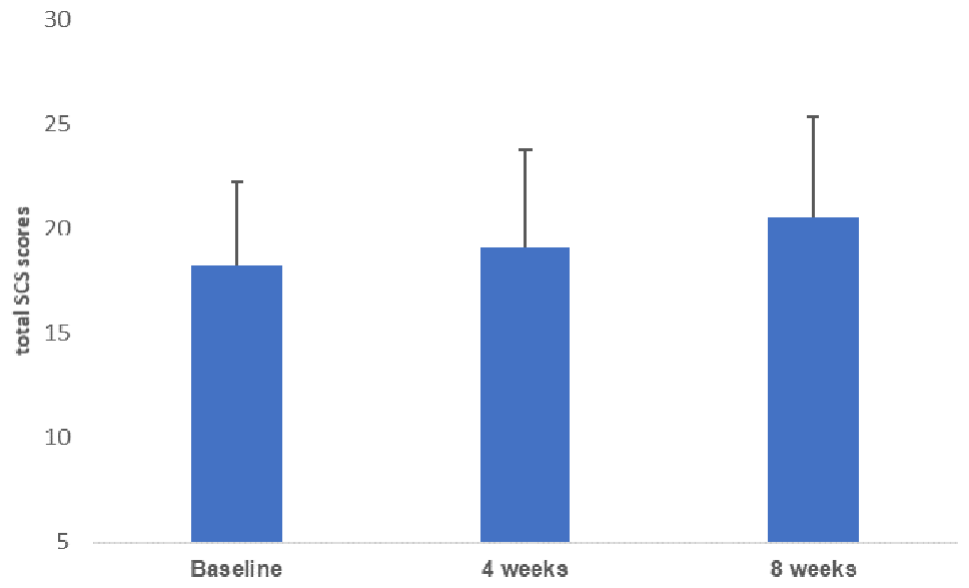
Changes in total self-compassion scale (SCS) scores from baseline to 8 weeks. The mean total self-compassion scale scores improved significantly from baseline to 8 weeks post-MBI. MBI, mindfulness-based intervention.

**Table 1** shows the demographic variables and baseline scores of the psychological tests. **Figure 1** depicts the change in the mean of total self-compassion scale scores over time [mean = 18.3 (SD = 4.0) at baseline, 19.1 (4.7) at 4 weeks, and 20.6 (4.8) at 8 weeks], and there was a significant change across time (F = 23.3, *p* = 0.0001) with a significant difference between baseline and 8 weeks (Tukey’s *post hoc* test, *p* = 0.02).

**Table 1:** Participant Demographics and psychological findings.

	*n* = 57
Age	49.6 (11.6)
Sex	
Male	10 (18%)
Female	47 (82%)
Education	
High school	11 (19.3%)
Junior/vocational college	24 (42.1%)
Bachelor/master/doctor	22 (38.6%)
TEMPS-A	
Depressive	8.9 (3.2)
Cyclothymic	4.8 (4.6)
Hyperthymic	4.5 (3.5)
Irritable	2.8 (3.1)
Anxious	7.0 (6.2)
TCI	
Novelty seeking	20.5 (5.3)
Harm avoidance	18.1 (7.1)
Reward dependence	14.3 (3.7)
Persistence	4.4 (2.0)
Self-directedness	28.5 (8.6)
Cooperativeness	27.3 (5.8)
Self-transcendence	12.1 (6.8)
MMSE	29.4 (0.8)
IQ (JART)	108.8 (7.9)
YMRS	0.4 (0.9)
HRSD	2.6 (3.2)
PBI	
Paternal care	20.7 (9.3)
Paternal overprotection	12.0 (6.5)
Maternal care	23.0 (8.3)
Maternal overprotection	12.1 (7.2)
T-score (PIL)	46.6 (10.0)

Data are mean (SD) or number (%) unless otherwise indicated.

TEMPS-A, Temperament Evaluation of Memphis, Pisa and San Diego Auto-questionnaire; TCI, Temperament and Character Inventory; MMSE, Mini-Mental State Examination; IQ, Intelligence Quotient; JART, Japanese Adult Reading Test; YMRS, Young Mania Rating Scale; HRSD, Hamilton Rating Scale for Depression; PBI, Parental Bonding Instrument; PIL, Purpose in Life Test.

**Table 2** shows the associations between the change between baseline and 8 weeks in total self-compassion scale scores (Δself-compassion scale scores) and age, gender, and the scores on TEMPS-A, TCI, MMSE, IQ (JART), YMRS, HRSD, PBI, and the T-scores (PIL). As shown in **Table 2**, novelty-seeking (TCI) was significantly and negatively associated with Δself-compassion scale scores (r = −0.282, *p* = 0.035) and the T-scores (PIL) significantly and positively associated with Δself-compassion scale scores (r = 0.286, *p* = 0.033). Educational attainment was not significantly associated with Δself-compassion scale scores (r = 0.229, *p* = 0.087). The multiple regression analysis with Δself-compassion scale scores as the dependent variable and novelty seeking (TCI), T-scores (PIL), age, and educational attainment as independent variables revealed that Δself-compassion scale scores were significantly and negatively associated with novelty seeking (TCI) (β = −0.293, *p* = 0.023) and significantly and positively associated with T-scores (PIL) (β = 0.271, *p* = 0.037). However, the associations with age and educational attainment were not statistically significant (**Table 3**).

**Table 2:** Associations of total self-compassion scale scores with relevant factors.

	Total self-compassion scale at baseline	Total self-compassion scale at 4 weeks	Total self-compassion scale at 8 weeks	Δ Self-compassion scale
Age	0.242	0.270*	0.222	0.068
Sex	0.192	0.236	0.302*	0.138
Education	−0.119	−0.043	0.053	0.229
TEMPS-A				
Depressive	−0.435*	−0.474***	−0.413**	−0.086
Cyclothymic	−0.410**	−0.368**	−0.303*	0.065
Hyperthymic	0.046	0.125	0.176	0.200
Irritable	−0.486***	−0.438**	−0.396**	0.015
Anxious	−0.477***	−0.430**	−0.466***	−0.101
TCI				
Novelty seeking	0.097	0.069	−0.097	−0.282*
Harm avoidance	−0.322*	−0.344**	−0.342*	−0.145
Reward dependence	−0.103	−0.081	−0.148	−0.101
Persistence	−0.178	−0.186	−0.062	0.099
Self-directedness	0.384**	0.447***	0.436**	0.225
Cooperativeness	0.174	0.210	0.221	0.159
Self-transcendence	0.006	0.053	0.061	0.108
MMSE	0.010	−0.098	−0.025	−0.007
IQ (JART)	0.160	0.223	0.219	0.110
YMRS	0.010	−0.032	−0.049	−0.081
HRSD	−0.279*	−0.249	−0.371**	−0.206
PBI				
Paternal care	0.139	0.182	0.204	0.102
Paternal overprotection	−0.180	−0.179	−0.222	−0.080
Maternal care	−0.144	−0.129	−0.141	−0.024
Maternal overprotection	−0.126	−0.066	−0.066	0.077
T-score (PIL)	0.579***	0.626***	0.660***	0.286*

∗p < 0.05.

∗∗p < 0.01.

∗∗∗p < 0.001.

**Table 3:** Multiple regression analysis showing the effects of age, educational attainment, novelty seeking, and purpose in life score on change in self-compassion during an 8-week mindfulness-based intervention (*n* = 57).

Variable	Change in self-compassion scale scores between baseline and 8 weeks (Δ self-compassion scale)		
	β	*t*-Value	*p*-Value
Constant		−0.415	0.680
Age	0.085	0.657	0.514
Educational attainment	0.227	1.771	0.083
Novelty seeking (TCI)	−0.293	−2.338	0.023
T-score (PIL)	0.271	2.143	0.037

Adjusted R^2^ = 0.153, F = 3.443, p = 0.015.

As shown in **Table 2**, novelty seeking was not significantly associated with the baseline, 4-week, or 8-week total self-compassion scale scores, whereas the T-scores (PIL) significantly and positively associated with the baseline, 4-week, and 8-week total self-compassion scale scores.

As sub-analyses, we calculated the percentage changes as ‘(self-compassion score at baseline minus self-compassion score at 8 weeks)/self-compassion score at baseline’ and performed correlation with novelty seeking (r = −0.249, *p* = 0.064) and PIL (r = 0.145, *p* = 0.285) by Pearson’s coefficient. Moreover, multiple regression analyses were performed with adjustment factors which were associated the percentage changes (*p* < 0.3). As a result, the percent changes were significantly and inversely associated with novelty seeking (β = −0.454, *p* = 0.003), but not with PIL (β = −0.079, *p* = 0.648), although this multiple regression analysis included 9 independent factors which were too many for 57 subjects to draw a conclusion. In any case, it should be noted that the correlation of the simple subtraction and the percent changes was highly significant in our subjects (r = 0.958, *p* = 0.0001).

## Discussion

These findings indicate that self-compassion significantly improved after the 8-week MBI and that improvement was significantly predicted by less novelty seeking and more PIL, suggesting that novelty seeking and PIL predict changes in self-compassion during this MBI. It should be noted that the novelty-seeking scores were not associated with total self-compassion scale scores, whereas the T-scores (PIL) were significantly and positively associated with the baseline, 4-week, and 8-week total self-compassion scale scores. This finding most likely means that self-compassion is closely related to PIL, but weakly related to novelty seeking. In concrete terms, because several of the self-compassion scale and PIL questionnaire items seem similar, self-compassion scale and PIL might somewhat conceptually overlap, partly explaining the significant positive association between the baseline and the 8 weeks post-MBI self-compassion scale scores. However, less novelty seeking is likely to actually predict Δself-compassion scale scores because the novelty-seeking scores were not associated with the baseline, 4-week, or 8-week total self-compassion scale scores.

With regard to the association between novelty seeking, mindfulness, and self-compassion, there is a report showing that individuals with attention-deficit/hyperactivity disorder (ADHD) had lower mindfulness and higher novelty seeking whereas those without ADHD had higher mindfulness and lower novelty seeking (30), suggesting a possibility that individuals with novelty seeking find it harder to disengage from spontaneous thoughts during a mindfulness exercise even if they do not suffer from ADHD, and that they may obtain less self-compassion as an effect of mindfulness.

The TEMPS-A and other character/personality scores of TCI (except novelty seeking) did not significantly predict the effects of MBI. In particular, hyperthymic temperament and cyclothymic temperament are widely accepted as premorbid temperaments of bipolar disorder, and the present findings suggest that such bipolarity do not predict the effects of an MBI, at least measured *via* change in self-compassion. Regarding educational attainment, in contrast to our previous report (1), the effects of MBI were not significantly predicted in our relatively small sample. The *p*-value of 0.083 implies the possibility that educational attainment might be a significant predictor for larger samples of participants.

It should be noted that we did not measure openness as a predictor of the responses to MBI. Since openness has been reported to be associated with more use of MBSR (8) and more mindfulness meditation (14), there remains a possibility that openness might have been selected as a predictor in this study if we measured it.

The limitation of the present study was a relatively small number of subjects which deterred a more sophisticated analysis of the pathways involved. Another limitation may be the likelihood of false positive effects due to multiple comparison and the exploratory nature of the analyses.

In conclusion, the present findings suggest that more PIL and less novelty seeking predict improvements in self-compassion during MBIs., and that apparently healthy individuals with more PIL and/or less novelty seeking can increase their self-compassion with the training of MBIs.

## Data Availability Statement

The raw data supporting the conclusions of this article will be made available by the authors, without undue reservation, to any qualified researcher.

## Ethics Statement

The studies involving human participants were reviewed and approved by the Oita University Ethics Committee. The patients/participants provided their written informed consent to participate in this study.

## Author Contributions

MA, TT, NK, and AS planned this study. MA, NK, and AS were actual working research team members. KH, MS, HH, KK, and NI discussed the results with the actual working members. MA and TT mostly wrote the manuscript; the other authors supported the study and checked the manuscript. All authors read and approved the final manuscript.

## Conflict of Interest

The authors declare that the research was conducted in the absence of any commercial or financial relationships that could be construed as a potential conflicts of interest.
